# Overcoming *Candida* biofilm resistance: targeting persister cells with probiotic-derived metabolites

**DOI:** 10.3389/frabi.2026.1767028

**Published:** 2026-02-24

**Authors:** Priyanka Debta, Binaya Krushna Sahu, Sudipta Kumar Patra, Fakir Mohan Debta, Ekagrata Mishra, Sujogya Kumar Panda

**Affiliations:** 1 Department of Oral Pathology and Microbiology, Institute of Dental sciences (IDS), Siksha ‘O’ Anusandhan (Deemed to be University), Bhubaneswar, Odisha, India; 2 Center for Biotechnology, Siksha ‘O’ Anusandhan (Deemed to be University), Bhubaneswar, Odisha, India; 3 Department of Orthopedics, Kalinga Institute of Medical Sciences, Bhubaneswar, Odisha, India; 4 Department of Oral Medicine and Radiology, Sriram Chandra Bhanja (S.C.B.) Dental College and Hospital, Cuttack, Odisha, India

**Keywords:** antifungal resistance, *Candida* biofilms, persister cells, postbiotics, probiotic-derived metabolites

## Abstract

*Candida* biofilms pose a significant complication in clinical settings due to antifungal drug tolerance and the presence of persister cells. Biofilm-mediated resistance is influenced by several associated factors, including the high density and extracellular matrix characteristics of the biofilm, metabolic downregulation, efflux pump activity, and stress-response signaling pathways, which ultimately diminish drug permeability and effectiveness. Within biofilms, persister cells form a small subpopulation of cells with unique phenotypic traits that enable them to survive lethal antifungal exposure and promote the recurrence of infection. Failure of antifungal treatments in eliminating biofilm and their resilient communities suggests a need for new, adjunct treatment options Recent findings have highlighted the therapeutic potential of probiotic-derived metabolites for inhibiting certain aspects of biofilm behavior and survival. These postbiotic compounds could offer a multi-faceted, low-toxicity treatment approach that may be used as an adjunct with existing antifungal therapies. Future investigations incorporating mechanistic studies, biofilm models, and drug product development for metabolite formulations could lead to a new treatment strategy for persistent *Candida* infections.

## 1 Introduction

*Candida* species are ubiquitous fungal commensals of the human mucosa that can become opportunistic pathogens, leading to a wide range of infections from superficial mucocutaneous overgrowth to life-threatening disseminated candidiasis (Kojic and Darouiche, 2004; Nett and Andes, 2020). A virulence trait contributing to many of the difficult-to-treat clinical infections caused by *Candida* is its ability to form biofilms, i.e., structured microbial communities that are attached to biotic or abiotic surfaces and embedded in an extracellular polymeric matrix (Ramage et al., 2005; Gulati and Nobile, 2016). Biofilm formation is particularly clinically relevant as it often forms on indwelling medical devices (e.g., vascular catheters, urinary catheters, prosthetic valves, endotracheal tubes) as well as on mucosal surfaces and surgical sites. Device-associated candidemia is associated with significant morbidity and mortality (Kojic and Darouiche, 2004; Nobile and Johnson, 2015). Biofilm cells exhibit collective phenotypes that differ from planktonic cells including altered metabolism, increased stress response, altered cell surface properties, and the secretion of a protective extracellular matrix (ECM) creating a niche environment, together all these factors contribute to their enhanced tolerance to antifungal therapy and immune clearance (Fanning and Mitchell, 2012; Cavalheiro and Teixeira, 2018; Nett and Andes, 2020; Atriwal et al., 2021).

The multifactorial antifungal tolerance of *Candida* biofilms is attributable to several contributions. The biofilm ECM contains polysaccharides, such as mannan-glucan complexes, extracellular DNA, proteins, and extracellular vesicle cargo, that together impede fungal penetration and may sequester antifungals, leading to reduced effective concentration at the cell surface (Roy and Gow, 2023; Nett and Andes, 2020). In addition, cells in mature biofilms exhibit altered gene expression patterns, with up-regulation of drug efflux pumps and stress-response pathways (heat-shock proteins, calcineurin signaling), metabolic shifts resulting in micro-niches (areas with hypoxia or nutrient limitation) that diminish activity from fungicidal drugs (Ramage et al., 2005; Robbins et al., 2011; Fanning and Mitchell, 2012). Adaptation at the community level, rather than individual resistance mutations, continues to explain why biofilm-associated infections do not respond to standard therapies, often requiring device removal or a combination of therapies for cure (Nobile and Johnson, 2015; Cavalheiro and Teixeira, 2018).

An additional clinically relevant phenomenon that occurs in biofilms, in some conditions, is the presence of persister cells. Persisters are phenotypic variants of an isogenic population that can tolerate, for a limited period, exposures to very high concentrations of antimicrobial agents, including antifungals, without developing inheritable resistance mutations. The earliest definitive experimental evidence of antifungal persisters in *C. albicans* biofilms was presented by LaFleur and colleagues, who demonstrated biphasic killing kinetics in biofilms exposed to amphotericin B or chlorhexidine. They reported on a small subpopulation that survived lethal exposures and then reseeded growth in exposed biofilms (LaFleur et al., 2006). The concept of lower viability subsequently continued to expand and become more nuanced. In many bacterial biofilms, persisters are considered a potential reservoir for relapse and the eventual emergence of genetic resistance. The same holds for fungal infections, particularly in relation to the potential for repopulation in biofilms depleted of an antifungal agent (Lewis, 2010). Persister cell biology in *Candida* is complex and is not universal. Some studies suggest that *C. albicans* biofilms rarely contain cells that resist the above products, indicating the formation of persistors is complex, strain-specific, and reliant on experimental conditions (Denega et al., 2019). Together, these data indicate that while persister cells likely contribute to treatment failure in some settings, they are one of several interacting mechanisms, alongside matrix sequestration, efflux activity, and metabolic heterogeneity, that produce the high tolerance of *Candida* biofilms.

Considering the limited antifungal classes available (azoles, polyenes, echinocandins) and the clinical challenges of eliminating biofilm-associated candidiasis, alternative or complementary treatment strategies are becoming of interest. Probiotic-derived metabolites (often termed “postbiotics” or “cell-free supernatants from probiotics”) are a class of interest. Bacterial probiotics (*Lactobacillus/Lactiplantibacillus* spp.) and probiotic yeasts (*Saccharomyces boulardii*) are capable of secreting organic acids (lactic acid, acetic acid, a variety of short-chain fatty acids), fatty acids (medium-chain fatty acids, like capric acid), and bacteriocin-like peptides and other small molecules that can suppress adhesion, hyphal transition, biofilm formation, and planktonic growth of *Candida in vitro* (Murzyn et al., 2010; García-Gamboa et al., 2024; Noverr and Huffnagle, 2004). Mechanistic studies indicate that some metabolites from probiotics downregulate hypha- and adhesion-associated genes (HWP1), disrupt membrane integrity, acidify the microenvironment, and interfere with quorum sensing and matrix production (Jiang et al., 2016; Ribeiro et al., 2017). Systematic reviews of postbiotics and other mechanistic studies extend the notion of characterized microbial metabolites as adjuncts that are stable and safe, avoiding some of the potential risks of live-microbe therapy, especially in immunocompromised patients (Malagón-Rojas et al., 2020; Zdybel et al., 2025; Prajapati et al., 2023; Aguilar-Toalá et al., 2021).

Recent epidemiological studies demonstrate substantial species-specific and geographic variability in antifungal resistance across different forms of candidiasis, as summarized in **Table 1**. Data from national surveillance programs and multicenter studies indicate that non-*albicans Candida* species, particularly *Candida glabrata*, *C. tropicalis*, *C. parapsilosis*, and *C. auris*, exhibit higher and more variable resistance rates compared with *C. albicans*, especially in cases of candidemia and other invasive infections. Resistance patterns also differ markedly by antifungal class and region; for example, echinocandin resistance in *C. glabrata* bloodstream isolates remains relatively low in the United States but shows an increasing trend, underscoring emerging therapeutic challenges. Collectively, these findings highlight the dynamic and region-dependent nature of antifungal resistance in *Candida* species and emphasize the need for continuous surveillance and alternative or adjunctive treatment strategies to manage resistant infections (Arendrup & Patterson, 2017; Pfaller et al., 2019; Nguyen, and Ren, 2025; Kaur et al., 2016). In parallel with these resistance trends, the global population of immunocompromised individuals, including patients with malignancies, transplant recipients, individuals with HIV infection, and those receiving immunosuppressive therapies, is steadily increasing, further amplifying the clinical burden and therapeutic complexity of resistant candidiasis (Sahu et al., 2026a; Sahu et al., 2025; Mallick et al., 2025; Das et al., 2026; Sahu et al., 2026b). Consequently, resistance rates are disproportionately higher among non-*albicans Candida* species in these vulnerable patient populations.

**Table 1:** Species-specific antifungal resistance patterns in different forms of candidiasis across geographic regions.

Reference	Region	Clinical context	Species	Resistance findings
Achilonu et al., 2025	Global	MDR *C. auris*	*C. auris*	Fluconazole resistance ~92.5%, voriconazole ~49%, amphotericin B ~51%
Baniodeh et al., 2024	Palestine	Various candidiasis	*C. albicans, C. glabrata, C. tropicalis, C. parapsilosis*	High frequency of NAC species which were often more resistant to antifungal agents
Bilal et al., 2022	Mainland China	Candidemia	*C. albicans, C. glabrata, C. tropicalis, C. parapsilosis*	Fluconazole susceptibility: *C. glabrata* ~79.4%, *C. tropicalis* ~77.95%
Kajihara et al., 2022	Japan	Candidemia	*C. glabrata, C. tropicalis, C. krusei*	Relatively high azole & micafungin resistance in non-albicans species
Verma et al., 2021	India	Candidiasis	*C. albicans, C. glabrata, C. tropicalis, C. parapsilosis*	NAC resistance high; *C. glabrata* (~20.69%), *C. albicans* (~10.34%)
Odoj et al., 2024	Europe	Candidemia	*C. glabrata, C. parapsilosis*	Some countries with 80–100% resistance in specific species
Yamin et al., 2022	Global	Candidemia	*C. parapsilosis*	Fluconazole resistance ~15.2%
CDC, 2024	USA	Candidemia	*C. glabrata*	Echinocandin resistance ~2% but rising
Osaigbovo et al., 2024	Africa	*C. auris* infections	*C. auris*	~91.3% fluconazole resistance; amphotericin B ~20.5%

Importantly, treatment efficacy against *Candida* biofilms is highly strain- and species-dependent (Gulati and Nobile, 2016; Nett and Andes, 2020). Clinical and laboratory studies have demonstrated substantial variability among *C. albicans* isolates, as well as between *C. albicans* and non-*albicans Candida* species, in terms of biofilm architecture, extracellular matrix composition, efflux pump expression, stress-response activation, and persister cell formation (Gulati and Nobile, 2016; Denega et al., 2019; Nett and Andes, 2020). These differences directly influence susceptibility to conventional antifungals and adjunctive strategies, including probiotic-derived metabolites (Kaur and Nobile, 2023). As a result, therapeutic responses observed in one strain or species cannot be universally extrapolated, underscoring the need for strain-inclusive evaluation when assessing anti-biofilm and anti-persister interventions (Denega et al., 2019; Kaur and Nobile, 2023).

The aim of this review is to summarize recent mechanistic and clinical evidence about how the combination of *Candida* biofilms and persister cells leads to antifungal tolerance, and the emerging literature on probiotic-derived metabolites as anti-biofilm and anti-persister therapy, emphasizing the mechanistic data, advantages and limitations of existing *in vitro* and *in vivo* studies, and potential future “postbiotic” therapies for adjunctive treatment.

## 2 Modulation of *Candida* biofilm resistance and virulence mechanism

*Candida* biofilms are characterized by the presence of an ECM, a dynamic, self-formed structure of macromolecules that encases sessile cells and alters the microenvironmental parameters of the biofilm community. Biochemical and compositional analyses of multiple *Candida* species indicate that the ECM has a high composition of polysaccharides, notably α-mannans and branched α-1,6/α-1,2 mannan and some β-glucan species, notably β-1,6 glucan, extracellular DNA (eDNA), and a diverse protein repertoire (enzymes and structural proteins) as well as some lipids and extracellular vesicle cargo (Pierce et al., 2017; Nett and Andes, 2020). The components of the ECM will vary in stoichiometry during different stages of biofilm development and differ among species; however, their relative presence creates a hydrated, viscoelastic gel that serves to stabilize intercellular connections, promote adhesion to surfaces, and sequester factors from both the host and/or other microbes. Recent reviews and experimental studies suggest that the ECM is not simply a passive scaffold: polysaccharides of the matrix and matrix-associated proteins actively bind small molecules and drugs, and matrix-derived extracellular vesicles provide additional enzymatic and structural factors that stabilize the integrity of the matrix (Roy and Gow, 2023; Pierce et al., 2017). Because of these properties, the matrix both physically slows the diffusion of antifungal agents and chemically interacts with them (for example, binding azoles or polyenes), thereby reducing the effective antifungal exposure to cells embedded deep in the biofilm (Nett and Andes, 2020; Roy and Gow, 2023).

Efflux pumps create an additional, complementary layer of tolerance associated with the existence of biofilms. Gene profiling and activity studies indicate that members of two major families of transporters, ATP-binding cassette (ABC) transporters (CDR1, CDR2) and the major facilitator superfamily (MFS, e.g. MDR1), are upregulated in the early biofilm community. However, persistence can occur in the mature biofilm (Atriwal et al., 2021). Upregulation may occur prior to any antifungal exposure, emphasizing that the activation of efflux is an inherent biological feature of the biofilm lifestyle, rather than a strictly downstream response to drugs. Inhibition of efflux, an experimental paradigm, has demonstrated reduced biofilm tolerance in *in vitro* models. Recent reviews suggest that efflux-mediated drug export is an actionable target to sensitize biofilms to conventional agents (Ren et al., 2024; Kaur and Nobile, 2023). It is essential to acknowledge that efflux contributes differently to tolerance, depending on both the drug class (efflux being particularly informative for azoles) and the fungal species, based on their transporter repertoire and expression time course.

Cellular stress-response pathways represent another key mechanism of survival for biofilm cells challenged with antifungal drugs. Biofilm growth is linked to the activation of highly conserved stress-response signaling cascades, primarily the heat shock protein (Hsp) network (Hsp90/Hsp70 and their co-chaperones), the calcineurin signaling pathway, and downstream gene expression responses, all of which help maintain protein folding, cell wall integrity, and ionic homeostasis under drug- and host-imposed stressors (Gong et al., 2017; Cowen, 2009; Roy and Tamuli, 2022; Ren et al., 2024). Hsp90, in particular, serves to stabilize client proteins, including calcineurin, important for tolerating azole, echinocandin, and polyene fungicidal activity; either pharmacologic or genetically induced destabilization of Hsp90-calcineurin signaling enhances fungicidal activity against biofilms (Cowen and Lindquist, 2005; LaFayette et al., 2010; Singh et al., 2009; Gong et al., 2017; LeBlanc et al., 2020). Recent mechanistic work also demonstrates that stress-response activation is mechanistically linked to biofilm-specific signaling phenotypes, including matrix biogenesis, metabolic reprogramming, and oxidative-stress resistance and persistence formation, which shows that stress pathways protect both individual cells and function to support community-level responses (Ren et al., 2024; Wang et al., 2025; Robbins et al., 2011; Uppuluri et al., 2018). Together, these signaling systems represent central regulators of drug tolerance in *Candida* biofilms and are key targets for antifungal-sensitizing strategies.

Modified membrane and cell physiology, specifically modulation of sterol biosynthesis and membrane composition, further reduces susceptibility to drugs in biofilms. Upregulation or modulation of genes involved in the ergosterol biosynthetic pathway (ERG family), as well as modifications to sterol content, may alter the binding or activity of polyenes, and in the case of azoles, change the abundance of the target enzyme (lanosterol 14α-demethylase), which affects its activity. Biofilm cells also remodel the components of their plasma membrane lipid composition and cell wall architecture, which contribute to physical and biochemical changes that reduce drug uptake or increase tolerance to membrane-directed stress (Atriwal et al., 2021; Kaur and Nobile, 2023). These changes to membranes are often integrated alongside efflux and stress-response pathways, producing layered irregularities that contribute to tolerance.

Metabolic variation in biofilms, including areas of nutrient limitation, oxygen gradients, and slow-growing or metabolically quiescent subpopulations, plays a crucial role in determining the efficacy of drugs. Many antifungals exert activity during the specific metabolic state of active growth and/or divided cells; therefore, the cells in micro-niches with low metabolic activity or hypoxia can tolerate exposures to the agent, while planktonic cells proliferating in their exponential phase can be killed (Pierce et al., 2017; Wall et al., 2019). This metabolic downregulation is also important when generating and/or maintaining persister cells, which are transiently dormant, drug-tolerant phenotypes that can reseed biofilms when they are no longer under drug pressure. Together, the interactions in time and space of matrix-mediated sequestration, efflux, stress-response buffering, membrane remodeling, and metabolic quiescence produce the antifungal tolerance seen in clinical biofilms. This complexity explains why single-target therapies often fail and why a multi-modal or adjunctive approach is indicated to eradicate device-associated and mucosal biofilm infections. **Table 2** summarizes the central cellular, biochemical, and structural mechanisms contributing to antifungal tolerance in *Candida* biofilms.

**Table 2:** Detailed mechanisms of antifungal resistance in *Candida* biofilms.

Mechanism	Molecular/cellular components	Effect on antifungal susceptibility	Drug classes affected	References
Extracellular Matrix	β-1,3/1,6-glucans, mannan-glucan complexes, eDNA, matrix proteins, extracellular vesicles	ECM binds/sequesters drugs, slows diffusion into deeper layers, and protects embedded cells	Azoles, polyenes, echinocandins	Pierce et al., 2017; Nett and Andes, 2020; Roy and Gow, 2023
Efflux Pumps	CDR1, CDR2 (ABC family); MDR1 (MFS family)	Active export of azoles; increased pump expression in biofilms increases drug efflux	Mainly azoles	Atriwal et al., 2021; Ren et al., 2024; Kaur and Nobile, 2023
Stress-Response Pathways	Hsp90 chaperone, calcineurin, Crz1 transcription factor, heat-shock proteins	Stabilization of stress proteins; cell-wall repair; survival under antifungal stress	Azoles, echinocandins, polyenes	Cowen, 2009; Gong et al., 2017; O’Meara et al., 2017
Membrane & Ergosterol Remodeling	ERG genes, altered ergosterol content, and membrane fluidity regulators	Reduced binding of azoles/polynes; altered permeability; increased membrane rigidity	Azoles, polyenes	Kaur and Nobile, 2023; Atriwal et al., 2021
Metabolic Heterogeneity & Quiescence	Hypoxic zones, nutrient-deprived layers, slow-growers, dormant cells	Fungicidal drugs are ineffective against slow-growing cells; survival of metabolically inactive phenotypes	Mostly fungicidal drugs (polyenes, echinocandins)	Wall et al., 2019; Pierce et al., 2017
Matrix-associated Extracellular Vesicles	EV cargo: polysaccharides, enzymes, β-glucan modifying factors	Reinforce matrix structure; deliver materials for ECM synthesis	All antifungal classes	Zarnowski et al., 2014; Mathé, and Van Dijck, 2013
Cell-wall Remodeling Pathways	PKC–MAPK pathways, chitin synthase upregulation	Thickened cell wall reduces drug penetration; compensatory chitin increases echinocandin tolerance	Mainly echinocandins	LeBlanc et al., 2020; Robbins et al., 2011
Oxidative Stress Defense	SODs, catalases, glutathione pathways	Detoxify ROS generated by antifungals; enhance survival	Polyenes, some azoles	da Silva et al., 2021; Wuyts et al., 2018

ECM, Extracellular Matrix; eDNA, Extracellular DNA; EVs, Extracellular Vesicles; ABC, ATP-Binding Cassette; MFS, Major Facilitator Superfamily; CDR, Candida Drug Resistance; MDR1, Multidrug Resistance 1; Hsp90, Heat Shock Protein 90; Crz1, Calcineurin-Responsive Zinc-finger 1; ERG genes, Ergosterol Biosynthesis Genes; PKC, Protein Kinase C; MAPK, Mitogen-Activated Protein Kinase; SODs, Superoxide Dismutases; ROS, Reactive Oxygen Species.

## 3 Persister cells in *Candida* biofilms

*Candida* biofilms are composed of a unique population of phenotypic variants that survive the use of fungicidal agents but do not pass on their traits through hereditary means. They are considered a non-heritable group of persister cells because they do not contain stable mutations, unlike the resistant mutant forms of fungi, which result in an increased minimal inhibitory concentration (MIC). The persisters survive drug exposure by being transiently tolerant, metabolically altered, or dormant until removed from the drug treatment. Only in this state can persister cells maintain their population density during the course of treatment and rebuild their population numbers once treatment ceases (Lewis, 2010; LaFleur et al., 2006). The persistent state occurs in fungal biofilms and has been defined in terms of its clinical significance and application (Denega et al., 2019; Uppuluri et al., 2018; Delarze and Sanglard, 2015).

Experimentally, the presence of persister cells in *Candida* biofilms is most often inferred from biphasic killing curves: when mature biofilms are challenged with high concentrations of a fungicidal agent, a rapid initial kill reduces the majority of the population but is followed by a plateau or much slower kill rate in which a small surviving fraction persists despite prolonged exposure (Lewis, 2010; LaFleur et al., 2006). LaFleur et al.’s classic study demonstrated this biphasic response in *C. albicans* biofilms exposed to amphotericin B and chlorhexidine and identified surviving cells that could reseed biofilm growth after drug removal, the operational signature of persisters (LaFleur et al., 2006). Subsequent studies across *Candida* species and clinical isolates have reproduced biphasic kinetics in many, but not all, settings, and the size of the persister fraction is highly variable, depending on the strain, substrate, and growth conditions (Denega et al., 2019).

Instead of single-gene resistance mechanisms, molecular investigations of persister cells indicate coordinated transcriptional and metabolic reprogramming (Lewis, 2012). Differential regulation of genes involved in sterol biosynthesis (ERG1, ERG25) and cell-wall/matrix polysaccharide pathways (including β-1,6-glucan-associated genes like SKN1 and KRE1) has been reported in comparative expression studies and targeted analyses of biofilm subpopulations. These findings are consistent with altered membrane and cell-wall physiology in persister-enriched cells (Attavar, 2010). Additionally, persisters exhibit gluconeogenic flow and energy storage pathways (trehalose and glycogen buildup), which are metabolic indicators of a transition to a quiescent, stress-protected state (Kuczyńska-Wiśnik et al., 2015; Wuyts et al., 2018). These changes plausibly reduce the efficacy of drugs that rely on active metabolism or target ergosterol biosynthesis, while simultaneously helping persisters tolerate membrane- or ROS-mediated damage.

Persister survival is mainly dependent on stress-response mechanisms. Persisters are frequently shielded from drug-induced oxidative stress and apoptosis-like pathways by molecular chaperones (most notably Hsp90 and small heat-shock proteins), the calcineurin signaling pathway, and enhanced antioxidant defenses (superoxide dismutases and other ROS-detoxifying enzymes) (O’Meara et al., 2017; Gong et al., 2017; Li et al., 2021). In particular, Hsp90 stabilizes important signaling clients, such as calcineurin, allowing cells to mount efficient cell-wall and membrane stress responses; in several models, disruption of Hsp90 or calcineurin makes biofilm cells more susceptible to antifungal agents (O’Meara et al., 2017; Gong et al., 2017; Li et al., 2021). Furthermore, persisters frequently display upregulation of the ROS-detoxification machinery and stress-protective storage molecules (trehalose, glycogen), which together blunt the lethal effects of fungicide-induced oxidative damage and support recovery once the drug pressure subsides (da Silva et al., 2021; Kuczyńska-Wiśnik et al., 2015; Wuyts et al., 2018).

Despite strong evidence for persisters in numerous experimental systems, significant debates and methodological limitations persist in the field. Some of the earlier findings may represent heterogeneous drug penetration, transient microenvironmental drug gradients, or assay artifacts rather than actual phenotypic persistence because several meticulously controlled reports have failed to find a stable persister subpopulation in some *C. albicans* strains or under particular experimental protocols (Denega et al., 2019). Whether biphasic killing is observed depends on several factors, including inoculum, substrate (plastic *vs*. mucosal tissue), biofilm age, drug exposure schedules, and detection sensitivity. As a result, apparent disagreements in the literature frequently reflect experimental context rather than a single biological phenomenon (Wuyts et al., 2018). Consensus reviews emphasize that persister formation in *Candida* is highly context- and strain-dependent, and that rigorous assay standardization and single-cell analyses are needed to resolve outstanding discrepancies.

Clinically, persister cells are invoked as a possible explanation for relapsing device-associated infections, when removal of the biofilm source is often necessary for treatment, and recurring mucosal infections (such as recurrent vulvovaginal candidiasis). Although direct in-patient persister identification is still technically challenging, *in vivo* and ex vivo biofilm models (rodent catheter and denture models, tissue explants) replicate biofilm architecture and have been used to show treatment failure and regrowth consistent with persister-mediated relapse (Li et al., 2015; LaFleur et al., 2006). To prevent relapse, strategies targeting metabolic dormancy, stress-response buffering (Hsp90/calcineurin), ROS detoxification, or matrix-associated protection are being actively investigated as supplements to standard antifungal regimens (Wuyts et al., 2018; Robbins et al., 2011). The major physiological and molecular features of *Candida* persister cells are summarized in **Table 3**.

**Table 3:** Detailed characteristics of *Candida* persister cells.

Feature	Molecular/physiological basis	Effect on antifungal susceptibility	Associated pathways/biomarkers	References
Non-heritable Drug Tolerance	Persisters arise from transient metabolic/physiological states rather than stable ERG or drug-target mutations	Survive concentrations >100× MIC; can regrow after treatment stops	Phenotypic heterogeneity; transient downregulation of growth pathways	Lewis, 2010; Delarze and Sanglard, 2015
Biphasic Killing Kinetics	Majority population rapidly killed, small persistent subpopulation remains,plateau in killing curve	Leads to treatment failure despite high-dose fungicidal drugs	Classic persister phenotype indicator	LaFleur et al., 2006; Wuyts et al., 2018
Metabolic Dormancy/Slow Growth	decrease Glycolysis, increase gluconeogenesis; accumulation of energy-storage molecules (trehalose, glycogen)	Fungicidal drugs are ineffective on non-growing cells	Trehalose synthase, glycogen synthase upregulation	Kuczyńska-Wiśnik et al., 2015; Wuyts et al., 2018
Activation of Stress Response Pathways	Hsp90 protects key signaling proteins; calcineurin stabilizes cell-wall repair; antioxidants reduce ROS toxicity	Enhances survival under azoles, echinocandins, polyenes	Hsp90–calcineurin axis; SODs, catalases	Li et al., 2021; O’Meara et al., 2017; Gong et al., 2017
Oxidative Stress Resistance	Increased ROS-detoxification enzymes and redox buffering	Polyenes and some azoles generate ROS; persisters survive	SOD2, catalase (CAT1), glutathione pathways	da Silva et al., 2021
Cell-Wall & Membrane Remodeling	Increased chitin synthesis, altered lipid composition	Reduces drug entry; compensates for echinocandin damage	Chitin synthase, ERG gene modifications	Robbins et al., 2011
Strain- and Condition-Dependence	Persisters appear only in some *C. albicans* strains and specific biofilm models	Not all isolates form persisters experimental variability	Depends on substrate, nutrient conditions, and drug regimen	Denega et al., 2019
Matrix-Associated Protection	Persisters are often found deep inside biofilm matrix niches	ECM limits drug penetration, facilitates persister survival	β-glucan-rich ECM microenvironments	Pierce et al., 2017; Nett and Andes, 2020

MIC, Minimum Inhibitory Concentration; Hsp90, Heat Shock Protein 90; ROS, Reactive Oxygen Species; SODs, Superoxide Dismutases; CAT1, Catalase 1; ECM, Extracellular Matrix.

## 4 Therapeutic challenges and current strategies

### 4.1 Limitations of conventional antifungals and the need for biofilm-specific adjuncts

Examples of the current antifungal drug classes used in clinical practice include the azoles, such as fluconazole; the polyenes, including amphotericin B; the echinocandins, including caspofungin and flucytosine. These antifungal agents are active against many *Candida* species in their planktonic (free-floating) forms. However, there is a significant challenge associated with these antifungal agents in treating biofilms formed by *Candida*, particularly those that are associated with medical devices and mucosal surfaces. The nature of biofilms results in substantial alterations in how antifungal agents access the cells of the biofilm and how the biofilm cells respond to these agents. For example, the structure of the ECM sequesters antifungal agents and slows down the diffusion of the antifungal agent to the cells of the biofilm, the efflux pumps and stress response systems in the biofilm cells are markedly upregulated. Thus, the concentration of the antifungal agent that is exposed to the cells of the biofilm is considerably lower than the concentration of the same antifungal agent that would be active against the planktonic cells (Nett and Andes, 2020; Kaur and Nobile, 2023). These various community-level phenotypes that develop due to the biofilm lifestyle result in lower effective drug concentrations at the target site and lower efficacy of fungicidal mechanisms that require the biofilm cells to actively metabolize (e.g., azole antifungals targeting ergosterol biosynthesis). In the clinical arena, this translates into a much higher minimum biofilm eradication concentration (MBEC) than would be expected based on the planktonic MIC, a frequent occurrence of treatment failure or relapse when the device is still in place, and the need for removal of the device or extended length of time receiving combination therapy (Roy and Gow, 2023). Because the mechanisms that confer tolerance to antifungal agents are multifactorial and partly redundant, the use of single-agent therapy alone is often insufficient, thus motivating research to develop adjunctive, biofilm-targeted therapeutic strategies rather than relying on increasing the doses of existing antifungal agents (Kaur and Nobile, 2023; Roy and Gow, 2023).

### 4.2 Experimental approaches targeting persisters and biofilm tolerance

**Figure 1:**
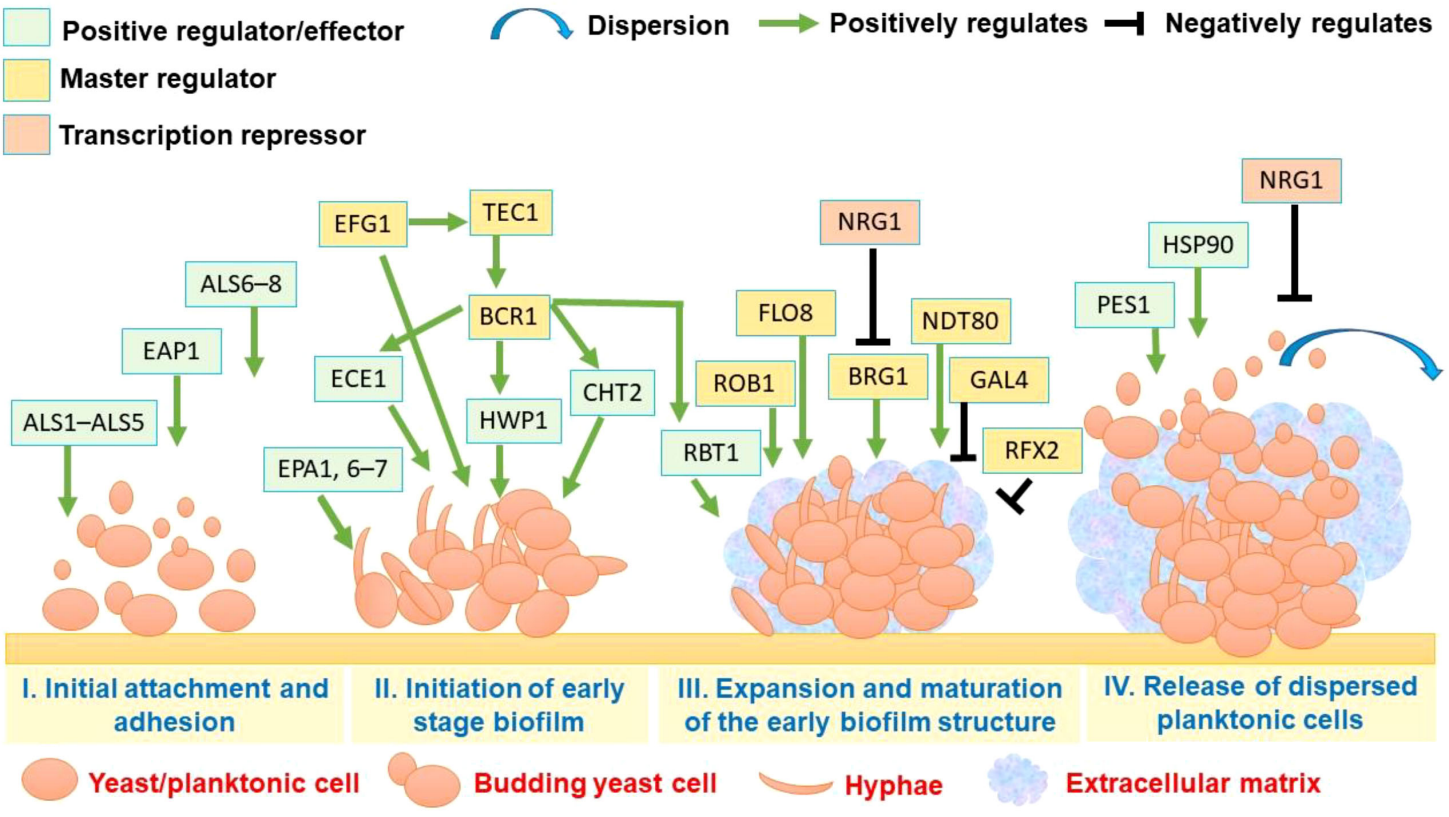
Regulatory network governing *C. albicans* biofilm development and dispersal. Enhanced Filamentous Growth protein 1 (EFG1), TEA/ATTS domain-containing transcription factor 1 (TEC1), Biofilm and Cell Wall Regulator 1 (BCR1), Agglutinin-Like Sequence protein 1 (ALS1), Agglutinin-Like Sequence protein 3 (ALS3), Agglutinin-Like Sequence protein 5 (ALS5), Agglutinin-Like Sequence protein 6 (ALS6), Agglutinin-Like Sequence protein 7 (ALS7), Agglutinin-Like Sequence protein 8 (ALS8), Enhanced Adherence to Polystyrene protein 1 (EAP1), Extent of Cell Elongation protein 1 (ECE1), Hyphal Wall Protein 1 (HWP1), Chitinase 2 (CHT2), Regulator of Biofilms 1 (ROB1), Repressed by TUP1 protein 1 (RBT1), Flocculation protein 8 (FLO8), Biofilm Regulator 1 (BRG1), Non-Dityrosine 80-like transcription factor (NDT80), Galactose Metabolism Regulator 4 (GAL4), Regulatory Factor X 2 (RFX2), Negative Regulator of Growth 1 (NRG1), Pescadillo homolog 1 (PES1), Heat Shock Protein 90 (HSP90).

Because both the persisters and other biofilm defenses are phenotypic, the means by which biofilms become resistant are methods that have been effective *in vitro* and in animal models to enhance the sensitivity of pathways that provide resilience against fungal stress, or to disrupt the structure of the biofilm matrix physically. An important approach to this area is the inhibition of the chaperone proteins involved in the fungal stress response, and their clients (the molecules they pick up and help keep stable). pharmacological inhibition of HSP90 (such as geldanamycin) and/or genetic perturbation of HSP90-calcineurin signaling increase the efficacy of azoles and echinocandins against biofilms and reduce the survival of persisters in experimental studies (Cowen, 2009; Robbins et al., 2011). Calcineurin inhibitors (such as tacrolimus and cyclosporin A) have also been shown to act in synergy with azoles to reduce resistance levels in specific biofilm assays; however, their immunosuppressive properties make their systemic use problematic and favour the use of calcineurin inhibitors in topical or device coating applications (Kaur and Nobile, 2023; Kiraz et al., 2025). These inhibitors of the stress pathways are also viewed as adjuvants because they most likely will not eradicate biofilm when used as a single agent. However, they can provide the breakdown of the stress buffer that allows persisters to be present at lower levels of antifungal activity, thereby maximizing the activity of azoles against biofilm (**Figure 1**).

In addition to inhibiting pathways, membrane- and peptide-based strategies are being considered that would allow for the direct permeabilization or destabilization of biofilm cells. Membranotropic Peptides (gH625 analogues) have been demonstrated to breach the ECM, allowing for improved antifungal penetration of compounds when combined with conventional treatments to kill persister-derived biofilms *in vitro*. These compounds are appealing due to their ability to work across multiple mechanisms (increased membrane disruption + increased antifungal uptake) and their potential to work in conjunction with standard antifungal medications; however, further optimization is required to ensure their stability/effectiveness *in vivo* and safety for use in the human body (Galdiero et al., 2020).

### 4.3 Matrix-disrupting adjuvants and enzymatic dispersal strategies

Enzymatic or chemical disruption of the ECM is a potential option for addressing drug sequestration limitations resulting from poor tissue penetration, and it has been extensively studied in the literature. Examples of matrix-degrading enzymes include glycoside hydrolases (including β-1,3/1,6-glucanases and mannanases), DNases (targeting extracellular DNA), and proteases. These enzymes can be used to metabolize ECM structural components (Wang et al., 2023) and reduce biofilm differentials by converting sessile cells into planktonic cells that are more susceptible to antimicrobial therapy. In addition, to date, studies with various bacterial and fungal systems have demonstrated that both enzyme-modified coatings and targeted enzyme on devices enhance the penetration of antifungals into biofilm structures and increase the killing power of antifungals against biofilms. For Candida infection *in vitro*, both β-glucanases and matrix-targeting agents reduced MBEC and enhanced the effects of echinocandins and polyenes (Tan et al., 2018; Wang et al., 2023). A similar pathway for achieving this goal is through the use of anti-biofilm enzyme coatings or adjuvant slow-release devices to prevent the formation of mature and poorly treatable biofilms. Limitations of this technology include enzyme stability, the immunogenic potential of enzymes, and the necessity of creating enzyme mixtures tailored to the specific matrix chemistry of each organism.

### 4.4 Gaps and future directions

#### 4.4.1 Need for better *in vivo* and clinically relevant models

Translational research is lacking in confirming the *in vitro* biofilm data (obtained using microtiter plates and flow devices) in the context of a human infection. Animal *in vivo* device studies (such as those on catheters and implants in rodents) were used to investigate the same types of host inflammatory responses (immune responses) and consider other factors that can significantly impact biofilm structures, matrix content, and antimicrobial susceptibility of biofilms (Nett et al., 2010). Many of these anti-biofilm agents that demonstrated efficacy *in vitro* will ultimately fail to work *in vivo* when tested against the immune system and/or other factors affecting the pharmacodynamics of the drug (such as drug clearance from the bloodstream and kinetics). Standardized, reproducible animal and ex vivo models that reflect polymicrobial niches, immune interactions, and clinically relevant exposure to anti-biofilm agents will enable the testing of adjunctive strategies against biofilm development prior to transitioning to clinical practice (Nett et al., 2010; Malinovská et al., 2023).

#### 4.4.2 Biomarkers and diagnostics for persister detection

Clinical biomarkers that accurately identify persister cells in human samples are not currently available. When attempting to diagnose either candidemia or biofilm infections and determine the severity of the infection, physicians primarily rely on laboratory cultures, molecular assays, and serum tests (e.g., β-D-glucan or mannan/anti-mannan), which indicate the total fungal burden but do not necessarily correlate with the existence of persistence-related reservoirs. There are many promising new technologies (e.g., single-cell transcriptomics, metabolic reporters, or molecular signatures) that could be utilized as candidate biomarkers for persistence-enriched states; however, these technologies still require controlled clinical studies for further validation and optimization (Chen, 2025; Wang et al., 2025). If implemented, the use of these biomarkers will lead to better utilization of adjunctive agents targeting persistence, as well as improved ability to monitor the risk of relapse.

#### 4.4.3 Strategies to prevent persister formation and enable eradication

Emergence of persistent fungi can be prevented through methods that support early, proactive intervention rather than waiting until established resistance has developed. There are several potential prevention strategies: (1) combining standard antifungals with other agents that affect stress response pathways, such as Hsp90/calcineurin inhibitors and membrane-active peptides, within a single treatment regimen. This approach aims to eliminate any chance of survival for persisting fungal cells by providing an environment that does not favour their survival and proliferation; (2) introducing prophylactic coatings on medical devices that inhibit adherence and the development of biofilms on implanted devices; (3) employing metabolic stimulants (also called metabolic potentiators) to move dormant cells past their non-drug-susceptible state to a drug-susceptible state immediately following exposure to an antifungal drug; and (4) employing rationally designed postbiotics, defined as products derived from probiotic work (e.g., probiotics) and created specifically to target both the inhibition of adhesion or the transition of yeast to filamentous hyphal forms, as well as sensitizing biofilm-forming cells to the effects of antifungal drugs. Multiple studies have provided proof of principle for the effectiveness of combination therapies (e.g., Hsp90 inhibition plus azole antifungals, peptides plus amphotericin B), as well as *in vitro*/future animal models demonstrating that these therapies can effectively eradicate persistent biofilms. However, further work is necessary to determine the optimal dosing, delivery route, and safety for these treatments before they can be used clinically (Galdiero et al., 2020; Robbins et al., 2011; Kaur and Nobile, 2023).

## 5 Probiotic-derived metabolites: therapeutic potential

In *in vitro* studies and increasingly in preclinical studies, numerous types of metabolites (secretory or chemically diverse) secreted by yeasts and probiotics include short- and medium-chain fatty acids, organic acids (e.g., lactic acid and acetic acid), biosurfactants, and small-sized aromatic compounds. Demonstrate antifungal and anti-biofilm effectiveness against the increasing prevalence of *Candida* spp. In early research and well-referenced studies, *Saccharomyces boulardii* culture filtrate (containing medium-chain fatty acids, with capric acid (C10:0) as the main fatty acid) has demonstrated the following: inhibiting hyphae formation, promoting hyphal transition, decreasing *C. albicans*’ adherence, and lowering biofilm formation by inhibiting virulence-related genes (HWP1 and CSH1) (Suchodolski et al., 2021). Multiple, later studies from all five genera of probiotic bacteria have confirmed and expanded on these findings, including culture filtration (“post-biotic”) of Lactobacillus spp. (including *L. rhamnosus, L. casei, L. acidophilus*, and *L. plantarum*) demonstrated that these products were capable of inhibiting the initial phases of *C. albicans* biofilm formation and to impart a reduction in adhesion to non-living surfaces and to inhibit the yeast-to-hypha formation of *Candida*, a critical morphologic switch for the development of a well-structured biofilm Architecture. These inhibitory effects are observed both when probiotics are present as live cells and when only their secreted metabolites (postbiotics) are applied, indicating that soluble molecules alone can mediate a significant portion of the anti-*Candida* activity (Matsubara et al., 2016; García-Gamboa et al., 2024).

The degree to which probiotics affect various pathogens will depend on the type of probiotic strain, the developmental stage of the pathogens, and whether the strains have been previously exposed to their target pathogens. Laboratory-made products using *L. rhamnosus* and *L. casei* supernatants were effective in decreasing the amount of biofilm formed during the initial stages of *Candida* infection and preventing the growth of filamentous fungi (Matsubara et al., 2016; Song and Lee, 2017). For *Candida* infections caused by non-albicans species, both *L. gasseri* and *L. rhamnosus* disrupted the established biofilms (after 24 hours of growth) by decreasing the number of viable cells present in those biofilms (Tan et al., 2018). Certain types of probiotic products (e.g., defined *L. plantarum* postbiotic products) also show a similar level of efficacy against candidiasis infection in laboratory-developed (artificially created) mycotic (fungus) populations, and to some degree in mixed populations of *Candida* spp., suggesting that their effectiveness might be enhanced within larger mixed populations of varying microorganisms (García-Gamboa et al., 2024). Pediococcus-derived strains (e.g., *Pediococcus acidilactici* HW01) have also been shown to exhibit similar anti-*Candida* activities by disrupting biofilm growth and formation. However, the factors responsible for this inhibition are primarily due to the production of organic acids, rather than traditional peptide-type bacteriocins (Kim and Kang, 2019). Together, these studies indicate that the potential of commercially available probiotic secretomes to inhibit biofilms formed by multiple species of microorganisms is quite wide (there are likely many different strains in the family of probiotic bacteria that have inherent properties to inhibit the formation of biofilms within the same family). A detailed summary of key probiotic-derived metabolites, their mechanisms, and anti-*Candida* effects is presented in **Table 4**.

**Table 4:** Probiotic-derived metabolites and their anti-*Candida* activities.

Postbiotic/metabolite	Producing organism(s)	Mechanism of action	Effect on *candida* virulence traits	Effective against	Notes/additional insights	References
Lactic Acid	*Lactobacillus rhamnosus*, *L. casei*, *L. acidophilus*	Lowers environmental pH; inhibits yeast–hypha transition; disrupts membrane potential	Decrease hyphal formation, decrease adhesion, decrease early biofilm	*C. albicans*, *C. glabrata*	Drives acid stress to suppresses filamentation required for biofilm structure	Matsubara et al., 2016; Paniágua et al., 2021
Capric Acid	*Saccharomyces boulardii* (postbiotic filtrate)	Inserts into fungal membranes; increases permeability; interrupts hyphal morphogenesis	Decrease filamentation, decrease adhesion, decrease mature biofilm biomass	*C. albicans*	One of the best-characterized postbiotic antifungals	Murzyn et al., 2010
Short-Chain Fatty Acids	Lactic acid bacteria; gut probiotics	Metabolic uncoupling; inhibits respiration; modifies intracellular redox	Decrease growth rate, decrease biofilm thickness, decrease virulence	*Candida albicans*, *C. tropicalis*, *C. parapsilosis*	SCFAs modulate fungal metabolism and can synergize with antifungals	García-Gamboa et al., 2024; Pedro et al., 2023
Biosurfactants	*Lactobacillus plantarum*, *L. rhamnosus*, *L. reuteri*	Reduce surface tension; inhibit adhesion to abiotic/biotic surfaces; disrupt cell–cell aggregation	Decrease adhesion, decrease initial colonization, decrease biofilm establishment	*C. albicans*, *C. tropicalis*	Highly effective in preventing early attachment on device surfaces	Poon and Hui, 2023; Song and Lee, 2017
Indole-3-Lactic Acid & Inosine Derivatives	*Lacticaseibacillus rhamnosus*, *L. acidophilus*	Modulate quorum sensing; inhibit fungal metabolic pathways	Decrease filamentation, decrease biofilm density	*C. albicans*	Strain-specific compounds; not all isolates produce equal levels	Spaggiari et al., 2024
Hydrogen Peroxide	Several *Lactobacillus* spp.	Oxidative inhibition; reduces fungal viability	Decrease growth, decrease biofilm, increase susceptibility to azoles	*C. albicans*, *C. glabrata*	Acts synergistically with organic acids	Vilela et al., 2015
Bacteriocin-like Peptides	*Pediococcus acidilactici*, *L. plantarum*	Pore-forming peptides; disrupt membrane integrity	Decrease viability of biofilm cells	*C. albicans*	Often active at low concentrations; good candidates for coatings	Kim and Kang, 2019

*Lactobacilli* produce organic acids (lactic, acetic and short-chain fatty acids) that help lower the local environment’s pH, thus making it difficult for filamentation and biofilm formation to occur. In addition, these organic acids may help “uncouple” the cellular metabolic activities of certain types of fungi from their ability to respire normally, resulting in a hypersensitivity to other stress agents (Paniágua et al., 2021; García-Gamboa et al., 2024). Medium-length chain fatty acids (for example, capric acid) can intercalate into fungal cell membranes, disrupting their normal hydrophobic properties and interfering with their ability to adhere to one another through their biophysical attachment sites. Furthermore, certain biosurfactant-producing probiotic microorganisms can lower the surface tension of liquid food products, thereby inhibiting the formation of stable membranes upon contact with non-biological surfaces (Murzyn et al., 2010). These observations have been confirmed by numerous studies, which have demonstrated that exposure to probiotics leads to the downregulation of genes associated with virulence and biofilm development in the fungi being exposed (Murzyn et al., 2010; Poon and Hui, 2023).

Additionally, multiple studies have demonstrated that the metabolites produced by probiotics can interact with those produced by other naturally occurring products or traditional antifungal medications. Several reported instances have demonstrated that combining honey and probiotic filtrates or low-dose antifungals with probiotic postbiotics leads to decreased biofilm biomass and reduced viability, as measured *in vitro*. The synergistic effect of probiotic postbiotics weakening certain defenses (adhesion, matrix, morphology) of multi-species biofilms would ultimately restore access to antifungals as well as potency against the pathogens (García-Gamboa et al., 2022). Mixed species biofilms, which typically contain both *Candida* and bacteria on either a mucosal surface or a device surface, are particularly effective in responding to the actions of probiotic filtrates. Data has shown that treatment with Lactobacilli supernatants or combinations of other probiotics results in lower polymicrobial biofilm thickness and lower matrix density than does treatment with a non-probiotic agent (Song and Lee, 2017; García-Gamboa et al., 2024).

Despite promising *in vitro* and ex vivo findings, significant limitations and gaps persist between research and clinical practice. The effectiveness of postbiotics is variable based on strain and method of preparation: Only some *Lactobacillus* strains or methods of preparing postbiotics produce antifungal activity that is comparable to that produced by other strains or methods of preparing postbiotics; and the concentrations that are effective *in vitro* are often greater than those that can be achieved *in vivo* without delivering the product directly to the desired site of action (via a targeted delivery system). The stability, standardization, and safety of concentrated postbiotics require assessment, particularly for individuals who are immunocompromised. The use of concentrated postbiotics in immunocompromised patients may be contraindicated, particularly when using probiotic forms of *Lactobacillus*. Coatings for devices or topical formulations that contain defined postbiotics are two methods of providing high concentrations at the desired site of action while also minimizing the risk of exposure to postbiotics in other locations (García-Gamboa et al., 2024; Murzyn et al., 2010). Most of the evidence regarding the ability of postbiotics to prevent or treat biofilm-associated candidiasis remains preclinical. Very few randomized clinical trials have evaluated the effect of postbiotics on the prevention/treatment of biofilm-associated candidiasis; however, the available data from both mechanistic and animal-model studies provide strong justification for developing these products for clinical use.

Available evidence indicates that probiotic-derived metabolites display both concentration-dependent and time-dependent antifungal activity against *Candida* species. Several studies demonstrate that increasing metabolite concentration enhances immediate inhibition of fungal growth, filamentation, and biofilm formation, whereas prolonged exposure, particularly at sub-inhibitory levels, progressively disrupts biofilm maturation, extracellular matrix integrity, and fungal viability. These pharmacodynamic patterns have been consistently observed across organic acids, fatty acids, and complex postbiotic preparations, highlighting that exposure duration is a critical determinant of anti-biofilm efficacy in addition to dose. These findings support the need for sustained delivery strategies when translating probiotic-derived metabolites into clinical or device-associated applications (Murzyn et al., 2010; Matsubara et al., 2016; Paniágua et al., 2021; García-Gamboa et al., 2024).

## 6 Challenges and limitations of probiotic approaches

### 6.1 Variability across strains and products

A significant barrier to developing effective treatments for *Candida* with postbiotics or probiotics is the high level of heterogeneity among different strains within the same species (Dube et al., 2020). For instance, isolates of *L. rhamnosus* or *L. plantarum* that have been classified as belonging to the same species exhibit a wide range of metabolite profiles, surface characteristics, and stress tolerance, depending on the conditions under which they were grown. *In vitro*, these differences manifest as differences in antifungal and anti-biofilm activity (Ansari et al., 2019; Spaggiari et al., 2024). Metabolomic investigations have demonstrated that even related species of lactic acid bacteria produce different levels of some small molecules (e.g., inosine and indole-3-lactic acid) that likely contribute to strain-specific antifungal activity against virulence factors (Spaggiari et al., 2024). Commercial probiotic products vary in terms of strain and often lack strain identifiers on their labels, making it challenging to replicate results and relate outcomes observed in clinical trials to their mechanistic basis (Ansari et al., 2019). Because the antifungal and anti-biofilm activities of probiotics and postbiotics depend on strain and growth conditions, recommendations based solely on the species of the organism are not appropriate for translating postbiotics into clinical products. Selecting the correct strain, controlling the growth and harvest conditions, and quantifying the chemical composition of the active fraction will be necessary for the successful translation of postbiotics into clinical products.

Similarly, variability among *Candida* strains further contributes to differences in treatment outcomes (Kaur and Nobile, 2023). Non-*albicans Candida* species often exhibit reduced susceptibility to azoles and distinct biofilm-associated tolerance mechanisms compared with *C. albicans* (Gulati and Nobile, 2016; Nett and Andes, 2020). Even within *C. albicans*, clinical isolates display heterogeneous responses to antifungals and postbiotic compounds due to strain-specific differences in matrix density, efflux transporter expression, metabolic state, and capacity to generate persister cells (Denega et al., 2019; Nett and Andes, 2020). Consequently, probiotic-derived metabolites and other adjunctive therapies may show differential efficacy across strains, reinforcing the importance of incorporating multiple clinical isolates and species in future translational and clinical studies (Kaur and Nobile, 2023).

### 6.2 Stability, formulation, and delivery challenges

While cell-free supernatants and probiotic metabolites (postbiotics) exhibit strong anti-*Candida* properties *in vitro*, maintaining effective concentrations of these compounds at their target sites *in vivo* presents many challenges. In addition to demonstrating safety and efficacy through large-scale clinical trials for postbiotics, other issues related to producing large quantities of postbiotics (including yield and stability during storage and subsequent processing) must also be considered. Production of short-chain fatty acids, medium-chain fatty acids, and small peptides, followed by formulation, requires stability and safety testing specific to the formulation method. Delivery systems, such as controlled-release gel formulations, impregnated coatings for medical devices, and mucoadhesive gels, have been developed to provide a means of delivering high levels of postbiotics to target sites while limiting systemic exposure; however, each delivery system must undergo individualized testing for stability and safety prior to being utilized in humans. Additionally, the development of processes for reproducibly producing specific postbiotics in industrial-scale quantities poses an ongoing regulatory and technical challenge for postbiotic research.

## 7 Emerging horizons and strategic research priorities

To accelerate the identification of postbiotics that can effectively target biofilm tolerance, a high-throughput discovery of metabolites and secretomes that act as anti-persisters will be necessary. There is growing evidence that a persister-based screen is possible, considering the development of a persister-based assay that can scale, as demonstrated by Petersen et al (Petersen et al., 2024). This really underscored the possibility of developing a drug-susceptibility assay that screens explicitly for compounds that are active against persister cells. They demonstrated this in the context of bacterial persisters, while addressing experimental parameters (i.e., starvation, exposure regimen) that enriched for persistent states, based on the design of their screen, which enabled the testing of large libraries of chemical compounds. In principle, similar persister-aware high-throughput screens could be adapted to investigate probiotic secretomes (i.e., cell-free supernatants and fractionated metabolomes) to systematically identify molecular motifs capable of killing or sensitizing fungal persisters, rather than solely inhibiting growth. Such screening would follow or combine automated fractionation (LC-MS guided), reliable miniaturized persister assays, and orthogonal readouts (viability, metabolic reporters, single cell imaging) to limit false positives that inhibit or kill only growing cells (Petersen et al., 2024).

Research in probiotic synthetic biology and metabolic engineering opens another promising avenue: it is possible to rationally engineer probiotics to overproduce specific antifungal metabolites, biosurfactants, or even secrete modified enzymes capable of degrading elements found within fungal matrices. Recent reviews and empirical studies have demonstrated that CRISPR/Cas, recombineering, and heterologous-expression toolkits enable the precise engineering of lactic acid bacteria and other probiotic chassis (Mugwanda et al., 2023; Ma et al., 2022). Engineered strains could establish localized factories (e.g., a vaginal gel or denture coating containing an engineered *Lactobacillus* capable of excreting the stable anti-biofilm peptide), or the secretome of the engineered strain could be purified and developed as a ready-defined postbiotic while mitigating the outset risks of using live microbes with the highest-risk patients (Yang et al., 2025; Ling et al., 2023).

Ultimately, screening and mechanistic investigations must occur in physiologically relevant models. Although simple microtiter-plate biofilm assays are ideal for initial triaging, they do not recapitulate the host factors (flow, shear, and adsorbed host proteins), polymicrobial interactions, immune effectors, or device materials. Achieving the translational goal will require the careful standardized use of ex vivo and *in vivo* models (catheter and denture rodent models, organoids and mucosal explants, and microfluidic “organ-on-chip” systems) that will permit controlled gradients and polymicrobial communities to be evaluated for both efficacy and host interactions with Candidate functional postbiotics and engineered probiotics (Nett et al., 2010; Carvalho et al., 2020; Andes et al., 2004; Řičicová et al., 2010). These models will further test whether Candidate metabolites penetrate the matrix, reach persister niches, or remain active in complex biological fluids.

### 7.1 Therapeutic development

Translating discovery into therapy will require defining product concepts and combination strategies. Two complementary paths are promising: (1) established postbiotic formulations - purified, chemically characterized metabolites, or metabolite mixtures that are formulated for topical or local use (gels, mouth rinses, vaginal suppositories, device coatings); and (2) adjunctive combination therapy where postbiotics are administered with existing antifungals to weaken biofilm defenses (inhibit hyphal transition, reduce matrix, suppress stress responses) and enable the use of lower antifungal doses to achieve eradication. Preclinical studies suggest a synergistic effect between membrane-active agents, stress-pathway inhibitors, and conventional antifungals (Galdiero et al., 2020; Iyer et al., 2022).

Designing the delivery system should focus on achieving high local concentrations with low systemic exposure factors, such as slow-release catheter coatings or mucoadhesive gels, to fully leverage activity against embedded persisters and minimize toxic effects on the host (Gao et al., 2024). Proof-of-concept clinical translation is being initiated, with registered clinical trials underway to assess postbiotic intravaginal preparations as adjunct therapy for vulvovaginal candidiasis (ClinicalTrials.gov NCT06474247). These trials demonstrate the regulatory and logistical pathways for the development of topical postbiotics. These pioneering clinical efforts will need to be scaled and coordinated with comprehensive pharmacokinetic/pharmacodynamic (PK/PD) studies that measure local metabolite concentrations, activity against biofilm samples ex vivo, and microbiome impacts to ensure efficacy and prevent dysbiosis (Pedro et al., 2023).

### 7.2 Regulatory and translational considerations

Safety, standardization, and manufacturability will determine whether valuable laboratory discoveries become viable medicines. At this moment, although regulatory clarity on postbiotics is developing, because they are chemically defined metabolites rather than live organisms, they are likely to follow a pathway similar to that of small molecule active pharmaceutical ingredients, which involves rigorous impurity profiling, stability, dose-range toxicology, and local tolerability studies, among other considerations. However, this also differs by jurisdiction and indication. Standardized assays for characterization (both untargeted and targeted metabolomics, activity-guided fractionation, identity/potency assays) are distinctly needed so that batches are reproducible, and mechanisms are traceable (Prajapati et al., 2023; Mishra et al., 2024).

Safety testing should match its intended route and population. Topical formulations for denture stomatitis or intravaginal gels will likely require local mucosal toxicity and microbiome-impact studies. For indwelling-device coatings, the focus needs to be on biocompatibility, durability, and leaching, all of which are assessed in a manner that mimics realistic flow conditions. For more at-risk patients (e.g., immunocompromised, catheterized), non-viable postbiotic formulations should be preferred over live probiotic formulations to lower the risk of sepsis and translocation. As a parallel to safety, ecological impact, i.e., whether postbiotics disrupt commensal bacterial communities or select for resistant opportunistic taxa, is also prime for agency consideration in approval and clinical application (Doron and Snydman, 2015; Prajapati et al., 2023).

The translational pathway would benefit from early thoughts from materials science, clinical, and regulatory experts to “co-design” delivery formats (e.g., special nanocomposite antimicrobial coatings that rapidly kill persisters on catheters) and to co-plan staged clinical trials with microbiological markers of success (i.e., MBEC reduction and no relapse) and not just surrogates of colonization. Integrated preclinical → to first-in-human → to indication-specific randomized clinical trials (RCTs) would be ideal candidates to combine mechanistic readouts (local drug/metabolite concentration, biofilm imaging, single-cell viability) with clinical readouts and, in advanced preclinical studies, local drug/metabolite concentrations.

## 8 Conclusion

*Candida* biofilms continue to pose a considerable therapeutic challenge due to their complex combination of biofilm-mediated resistance mechanisms and surviving persister cells that cause antifungal resistance and infection relapse. Recently, metabolites derived from probiotics have emerged as novel adjunctive agents that can inhibit adherence, reduce hyphal development, disrupt biofilm structure, decrease virulence, and potentially impact persister-like subpopulations. Although results are encouraging *in vitro*, challenges related to metabolite stability, barriers to delivery, as well as strain variability and safety issues, need to be addressed before considering clinical use. Continued mechanistic studies, improved biofilm and persister models, refined metabolite formulations, and carefully controlled clinical trials are necessary to fully realize the benefits of postbiotics in combination with integrated approaches for treating persistent *Candida* infections.
